# Effect of Gestational Diabetes on Fetal Growth Rate and Later Overweight in the Offspring

**DOI:** 10.1210/clinem/dgae428

**Published:** 2024-06-25

**Authors:** Magnus Leth-Møller, Adam Hulman, Ulla Kampmann, Susanne Hede, Per G Ovesen, Sine Knorr

**Affiliations:** Dept. of Clinical Medicine, Aarhus University, 8200 Aarhus N, Denmark; Dept. of Obstetrics and Gynaecology, Aarhus University Hospital, 8200 Aarhus N, Denmark; Steno Diabetes Center Aarhus, Aarhus University Hospital, 8200 Aarhus N, Denmark; Steno Diabetes Center Aarhus, Aarhus University Hospital, 8200 Aarhus N, Denmark; Dept. of Public Health, Aarhus University, 8000 Aarhus C, Denmark; Dept. of Clinical Medicine, Aarhus University, 8200 Aarhus N, Denmark; Steno Diabetes Center Aarhus, Aarhus University Hospital, 8200 Aarhus N, Denmark; Healthcare service for families, Aarhus Municipality, 8260 Viby J, Denmark; Dept. of Clinical Medicine, Aarhus University, 8200 Aarhus N, Denmark; Dept. of Obstetrics and Gynaecology, Aarhus University Hospital, 8200 Aarhus N, Denmark; Steno Diabetes Center Aarhus, Aarhus University Hospital, 8200 Aarhus N, Denmark; Dept. of Clinical Medicine, Aarhus University, 8200 Aarhus N, Denmark; Steno Diabetes Center Aarhus, Aarhus University Hospital, 8200 Aarhus N, Denmark

**Keywords:** childhood obesity, fetal growth, fetal programming, pregnancy, gestational diabetes, trajectories, gestational diabetes, childhood obesity

## Abstract

**Context:**

Children of women with gestational diabetes (GDM) are often born with a higher birthweight and have an increased risk of overweight during childhood. High fetal growth rate is also associated with being overweight in childhood.

**Objective:**

To examine excessive fetal growth rate as a mediator between GDM and overweight in the offspring.

**Methods:**

This was a longitudinal cohort study, using routinely collected data on children born between 2008 and 2014 in Aarhus, Denmark. Fetal biometrics were extracted from the patient records at Aarhus University Hospital and childhood weight from the health records at Aarhus Municipality Healthcare Service. We calculated growth trajectories for fetuses affected by GDM and for unaffected fetuses using cubic mixed model regression. We extracted individual fetal growth rate and estimated the contributing effect of fetal growth rate on the risk of being overweight in the 5- to 9-year-old offspring.

**Results:**

We included 6794 mother-child pairs, 295 with GDM. Fetal growth was higher in women with GDM from week 25, and the offspring had an increased risk of being overweight (odds ratio, 2.02 [95% CI, 1.44-2.84]). When adjusting for fetal growth rate in week 28, the effect attenuated by 15%, and to 1.10 (95% CI, 0.76-1.60) when further adjusting for prepregnancy body mass index.

**Conclusion:**

Pregnancies affected by GDM had higher fetal growth rate and the offspring had a higher risk of being overweight at age 5 to 9 years. Fetal growth rate in early third trimester was a mediator of up to 15% of this association, but prepregnancy body mass index contributed strongly as well.

Children of women with overweight or gestational diabetes (GDM) often become overweight (1, 2), and these children have a high risk of overweight in adolescence and adulthood (3), resulting in increased risk of lifestyle diseases (4). However, how the risk of being overweight is passed on to the offspring is far from clear. It most probably depends on multifactorial pathophysiological mechanisms, with genes, socioeconomics, and lifestyle all playing a part (5). Among the more well-known risk factors for overweight during childhood, is maternal overweight, GDM, and being born large-for-gestational age (2).

In more recent years, there has been increasing focus on intrauterine programming and fetal growth. Fetal growth has traditionally been evaluated by its final product, birthweight, but recent studies have shown that high fetal growth rate itself is associated with increased growth in infancy and overweight in childhood at ages 3 to 6 years (6-10).

At the same time, hyperglycemia in pregnancy has been shown to be associated with increased fetal growth (11, 12) and increased abdominal circumference (13). But little has been reported on how fetal growth might be a mediator between GDM and overweight in the offspring.

The aim of this study is to describe fetal growth rate in pregnancies affected by GDM compared to pregnancies with normal glucose tolerance (NGT) and to investigate if fetal growth rate could be a mediator in development of childhood overweight in offspring of women with GDM.

We hypothesized that children of mothers with GDM have a higher risk of becoming overweight and that part of this risk is mediated by increased fetal growth rate in pregnancies affected by GDM.

## Materials and Methods

### Study Design and Participants

The current study is a longitudinal cohort study using routinely collected data on children born in Aarhus, Denmark, from 2008 until April 2014, with follow-up until April 2019. The cohort comprises all children in Aarhus Municipality and contains health information, including weight and height. The information was collected by a health visitor at school consultations, which is offered to all children in Denmark. The participation rate to the program is not registered but a participation of approximately 95% has been estimated (14). If the child had not started school at the measurement time, the measurement has been taken at home by a specialty health nurse.

The study was approved by the Danish Patient Safety Authority (3-3013-2665/1) and The Danish Data Protection Agency (1-16-02-619-18). Ethical approval and patient consent were waived because of the epidemiological design and the size of the study population in accordance with the General Data Protection Regulation in Denmark.

We included all singleton pregnancies with live born children born at term (weeks 37-42). The women had to have at least 1 ultrasound scan in addition to the first trimester scan, with recorded estimated fetal weight (EFW) from gestational week 19 and onward and birthweight of the baby. We excluded women with type 1 or type 2 diabetes.

To estimate the main outcome (being overweight at school age), the children had to have a recorded weight and height at ages 5 to 9 years.

### Gestational Diabetes

In Denmark, risk factor-based screening is applied rather than universal screening for GDM. GDM is diagnosed based on the 2-hour 75-g oral glucose tolerance test (OGTT) if plasma glucose is ≥9.0 mmol/L after 2 hours (15).

Risk factors used in screening are pregestational overweight (body mass index [BMI] ≥ 27 kg/m^2^), family disposition for diabetes, previous birth of large baby (≥4500 g), and polycystic ovary syndrome. Women with previous GDM or 2 or more risk factors are offered screening with an OGTT in gestational weeks 10 through 20 and again in gestational weeks 24 through 28 (if the first OGTT was negative). Women with 1 risk factor or multiple pregnancies are offered an OGTT in gestational weeks 24 through 28. Furthermore, pregnant women are universally screened for glucosuria and, if positive at any point in pregnancy, they are offered an OGTT. These criteria remained unchanged throughout the study period, whereas the prevalence in Central Region Denmark increased from 2.4% in 2008 to 4.2% in 2014 (16).

The first-line treatment for GDM in Denmark is diet and lifestyle counseling. The women are advised to self-monitor blood glucose 6 times per day, and if they have continuously elevated glucose levels, defined as 2 or more measurements of glucose above 6 mmol/L before meals or above 8 mmol/L 1.5 hours after meals in a 2-week period, insulin treatment is initiated (17). Metformin is not used during pregnancy in Denmark.

### Fetal Growth Assessment

We accessed the Picture Archiving and Communications System (Astraia, Astraia Software GmbH, München, Germany) at Aarhus University Hospital. We extracted EFW, head circumference, femur length, and abdominal circumference, gestational age, and birthweight. If EFW was missing, it was calculated using the Hadlock III formula (18). We used data from all scans conducted from gestational week 19 until term and birthweight was used in continuation of the ultrasound-based fetal weights. As such, each pregnancy contributed with at least 2 measurements—1 ultrasound estimated fetal weight and a birthweight, and if more scans were performed these were included as well. If birthweight was missing, it was supplemented from the Danish Fetal Medicine Database.

Scans were performed by sonographers or trained medical doctors. Gestational age was confirmed at the week 12 scan. The assessment of growth rate is described in the statistical analysis section.

To further qualify fetal growth, we analyzed the abdominal circumference at weeks 19 through 24. We calculated *Z*-scores using the formula by Verburg et al (19) as a reference.

Sex-specific birthweight *Z*-scores were calculated using a Scandinavian reference population (20). Additionally, rates of small-for-gestational age and large-for-gestational age was calculated using cutoffs of birthweight <10th and >90th percentiles, respectively.

### Child Anthropometrics

Our main outcome was overweight at age 5 to 9 years. We used height and weight to calculate sex-specific BMI-for-age *Z*-scores using World Health Organization reference material and defined overweight as BMI-for-age *Z*-scores of more than 1 standard deviation as per the World Health Organization definition (21). If multiple measurements were recorded, we would use the latest.

### Covariates

Maternal prepregnancy weight, height, and BMI, smoking (yes/no), ethnicity (Caucasian or other), parity, gestational age at birth, and mode of delivery (vaginal or cesarean section) were extracted from the electronic records stored in Astraia. The records also provided information on diabetes diagnosis (type 1, type 2, or diet- or insulin-treated GDM).

### Statistical Analysis

Population characteristics were described as means and SD for continuous variables and as frequencies (%) for categorical characteristics.

#### Fetal growth

At first, we described fetal growth in women with GDM and women with NGT. We estimated cubic fetal growth trajectories using a mixed model regression with a linear, quadratic, and cubic term for time and included interaction terms with GDM diagnosis. Gestational age was centered to week 20 in all models. We included random effects for intercept and linear and quadratic terms. This model is referred to as Mixed Model 1. By adding the random effects to the fixed effects, we estimated the individual fetal growth trajectories.

To evaluate the effect of diabetes severity, we created Mixed Model 2, which was similar to Mixed Model 1, except we created 3 groups instead of 2: women with NGT, women with diet-treated GDM, and women with insulin-treated GDM.

Second, we wanted to assess the mediating effect of fetal growth rate on the association between GDM and overweight in the offspring. Therefore, we performed a 2-step analysis comprising step 1, which created a model from which we extracted the individual level fetal growth rate and step 2, which evaluated the effect of GDM and fetal growth rate extracted from step 1 on childhood overweight in a logistic regression model.

**Step 1**. We created a cubic mixed model identical to Mixed Model 1 but without interaction with GDM diagnosis (Mixed Model 3). We then estimated the individual fetal growth rate by combining the random and the fixed effects from Mixed Model 3.

**Step 2.** To evaluate the main outcome, childhood overweight, we used logistic regression to study the risk of overweight in children affected by GDM during fetal life compared to unaffected children. The individual fetal growth rates from Mixed Model 3 were then included in the logistic regression model to evaluate the effect on the estimate. We used fetal growth rates in week 28. Logistic models are reported unadjusted, adjusted for maternal age, parity, smoking, and further adjusted for prepregnancy BMI. Analyses was performed as complete case analysis.

### Supplementary Analyses

We conducted a supplementary analysis comparing baseline characteristics in healthy controls who had received only routine antenatal care compared to healthy controls who had additional ultrasound scans done. We compared maternal age, prepregnancy BMI, smoking status, ethnicity, parity, birthweight, birthweight *Z*-score, small for gestation age and large for gestational age rates, gestational age, and cesarean section rates between the 2 groups. We also compared those who were lost to follow-up with the children included in the cohort.

All data analyses were performed using R version 4.3.0 (2023-04-21) (R Foundation for Statistical Computing, Vienna, Austria). We used the r-package *nlme* (version 3.1.162) for mixed models.

## Results

### Study Population

**Figure 1.**
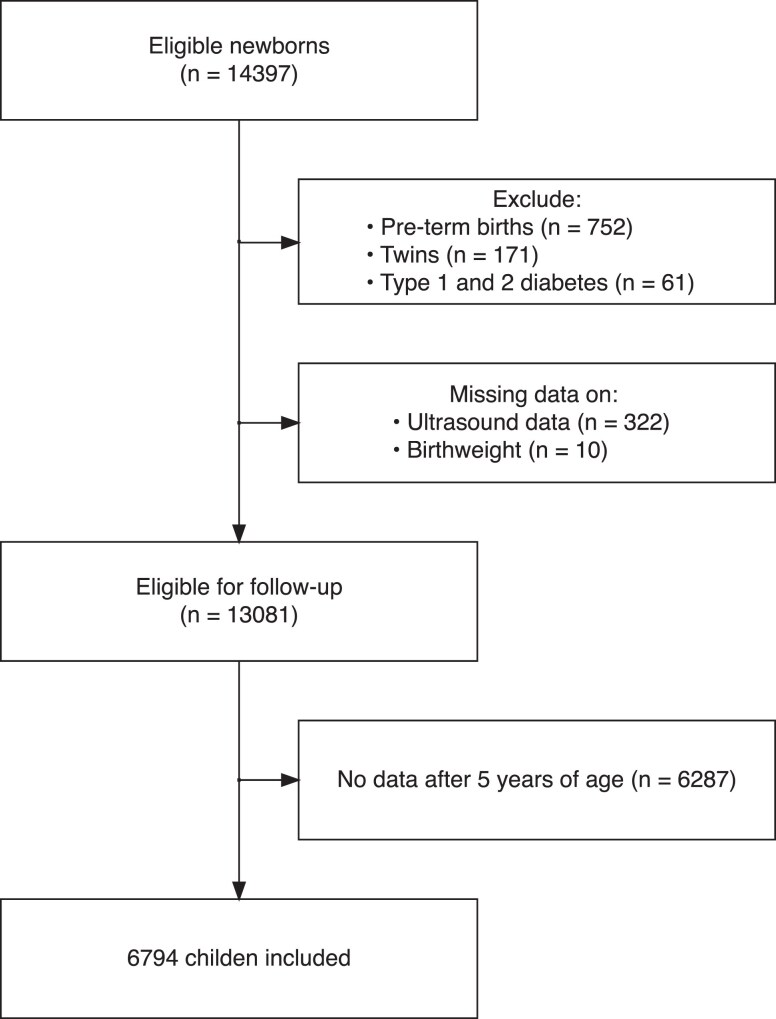
Flowchart of participant inclusion.

In total, 14 397 eligible newborns were identified, of which 13 081 met the inclusion criteria (Fig. 1). Of those, 6794 had weight and height measurements after the age of 5 years and were included in the analysis. A total of 295 children were born of mothers with GDM.

Table 1 shows the characteristics of the study population.

**Table 1.** Baseline characteristics

Characteristic	N	No diabetes, N = 6499^a^	GDM, N = 295^a^
Maternal age (y)	6794	30.5 (4.8)	32.6 (5.1)
Prepregnancy weight (kg)	6694	66.3 (13.1)	76.0 (17.7)
Height (cm)	6714	168.1 (6.5)	165.0 (7.6)
BMI (kg/m^2^)	6734	23.4 (4.2)	27.9 (6.3)
Smoking	6727	374 (5.8%)	14 (5.1%)
Ethnicity	6716		
Caucasian		5899 (91.6%)	233 (83.5%)
Other		538 (8.4%)	46 (16.5%)
Parity (nullipara)	5707	2450 (44.3%)	81 (44.5%)
GDM treatment	6794		
No		6499 (100.0%)	0 (0.0%)
Diet		0 (0.0%)	258 (87.5%)
Insulin		0 (0.0%)	37 (12.5%)
Fetal growth rate, week 28 (g/wk)	6794	175.6 (15.7)	184.2 (20.2)
Birthweight (g)	6794	3542.9 (466.6)	3565.2 (455.9)
Birthweight *Z*-score	6794	−0.1 (1.0)	0.3 (1.1)
Size at birth	6794		
AGA		5291 (81.4%)	230 (78.0%)
SGA		724 (11.1%)	19 (6.4%)
LGA		484 (7.4%)	46 (15.6%)
Abdominal circumference *Z*-score weeks 19-24	6557	0.0 (0.9)	0.3 (1.0)
Sex (boy)	6794	3218 (49.5%)	144 (48.8%)
Gestational age (days)	6794	281.2 (8.3)	276.0 (7.7)
Cesarean section	6722	1059 (16.5%)	82 (28.1%)
Exclusive breastfeeding	5447		
No breastfeeding		774 (14.7%)	47 (24.6%)
0-3 mo		903 (17.2%)	37 (19.4%)
More than 3 mo		3579 (68.1%)	107 (56.0%)
Number of ultrasound scans	6794	1.8 (1.2)	3.9 (1.5)
Age at BMI measurement. (y)	6794	7.2 (0.7)	7.4 (0.8)

Abbreviations: AGA, appropriate for gestational age; BMI, body mass index; GDM, gestational diabetes; LGA, large for gestational age; SGA, small for gestational age.

^*a*^Mean (SD); n (%).

### Fetal Growth

**Figure 2.**
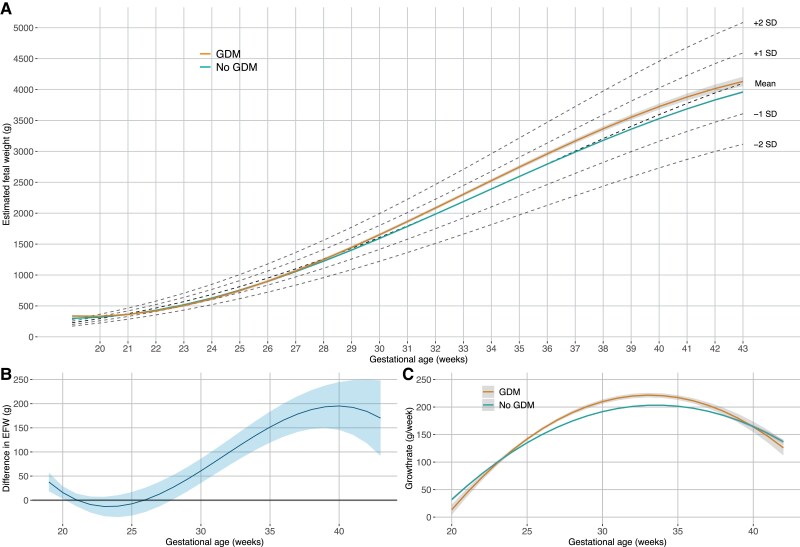
Fetal growth plots. (A) Fetal growth from week 20 until birth in pregnancies affected by gestational diabetes (GDM) and unaffected pregnancies with 95% CI (gray shading). Dashed lines show mean ± 2 SD using a Scandinavian reference material (20). (B) Fetal growth rate in pregnancies affected by GDM and unaffected. (C) Difference in fetal size in pregnancies affected by GDM compared to unaffected. Shaded area: 95% CI.

Fig. 2A shows the fetal growth in pregnancies affected by GDM and unaffected pregnancies. We found that pregnancies affected by GDM had a higher weekly growth rate from week 25 until week 38 (Fig. 2B and Supplementary Table S1 (22)), which resulted in a higher weight from week 28 and throughout pregnancy with a mean difference at week 40 of 195 g (95% CI, 146-245) (Fig. 2C). Estimated fetal weights are shown in Supplementary Table S2 (22). Mean abdominal circumference *Z*-score at weeks 19 through 24 was 0.0 SD in women without GDM and 0.3 in women with GDM.

#### GDM treatment

Among the pregnancies that were affected by GDM, 258 were diet treated and 37 were insulin treated. Fetal growth rate was higher in women with insulin-treated GDM than women with diet-treated GDM, resulting in a larger fetal size. At term (gestational age 40 + 0), children of women who were insulin treated were 324 g (95% CI, 175-473) larger compared to children of women with diet-treated GDM. Children of women with diet-treated GDM were 154 g (95% CI, 101-206) larger at week 40 compared to children not affected by GDM. For trajectories and coefficients see Supplementary Fig. S1 and Supplementary Table S3 (22).

### Childhood Overweight

Among children of mothers with GDM, 90 (30.5%) were overweight. Among children of mothers with NGT, there were 1095 (16.9%) with overweight. The odds ratio for overweight in children whose mothers had GDM during pregnancy compared to children of mothers with NGT during pregnancy (adjusted for smoking, maternal age, and parity) was 2.02 (95% CI, 1.44-2.84), see Table 2. When including individual fetal growth at week 28, the effect of GDM on childhood overweight in the 5- to 9-year-old offspring attenuated by 15% (adjusted OR: 1.72 [95% CI, 1.22-2.44]). When additionally adjusting for prepregnancy BMI, the OR for overweight in the offspring of women with GDM attenuated to 1.21 (95% CI, 0.84-1.75) before adjusting for fetal growth rate in week 28. There was only a slight attenuation when adjusting for fetal growth rate in this model (Table 2). Maternal prepregnancy BMI was associated with childhood overweight (OR: 1.13 for every unit change in BMI [95% CI, 1.11-1.14]).

**Table 2.** Odds ratio (95% CI) for overweight in the offspring of mothers with GDM (gestational diabetes) compared to offspring of mothers with normal glucose tolerance in models with and without fetal growth rate

Model	Without fetal growth	With fetal growth
Unadjusted, n = 6794	2.17 (1.68-2.80)	1.85 (1.43-2.41)
Adjusted for smoking, parity, age, n = 5697	2.02 (1.44-2.84)	1.72 (1.22-2.44)
Adjusted for smoking, parity, age, pre-pregnancy BMI, n = 5669	1.21 (0.84-1.75)	1.10 (0.76-1.60)

Abbreviation: BMI, body mass index.

In the secondary analysis exploring the effect of GDM treatment, the OR (adjusted for smoking, parity, and age) for overweight in the offspring of women with diet-treated GDM compared to unaffected children was 2.00 (95% CI, 1.40-2.87). For children of insulin-treated GDM, the odds ratio for overweight was 2.18 (95% CI, 0.82–5.77). When including individual fetal growth at week 28, the adjusted OR attenuated to 1.75 (95% CI, 1.22-2.53) for children of mothers with diet-treated GDM and to 1.51 (95% CI, 0.56-4.10) for children of mothers with insulin-treated GDM.

### Supplementary Analyses

Of the 6499 healthy women, 2913 (45%) had additional scans done. The women who received additional scans were similar to those who received routine care on age, prepregnancy BMI, smoking, and had similar birthweight *Z*-scores, albeit slightly smaller among women with additional scans. Most noticeably, the rate of small-for-gestational age was higher, whereas large-for-gestational age rates were similar. Also, cesarean section rate was higher. See Supplementary Table S4 (22). Those lost to follow-up had a slightly lower rate of GDM but were otherwise similar to those included in the cohort (Supplementary Table S5 (22)).

## Discussion

In this longitudinal study, we found that in pregnancies affected by GDM, fetal growth rate was higher from late second trimester compared to pregnancies with NGT, resulting in a larger EFW from early third trimester and throughout pregnancy.

Children of mothers with GDM had double the risk of being overweight, but this risk attenuated when adjusting for fetal growth rate, meaning that fetal growth rate might be a mediator between GDM and overweight in the offspring. Adjusting for prepregnancy BMI also markedly attenuated the effect of GDM on overweight offspring.

In women with GDM, the decreased insulin sensitivity leads to higher levels of both insulin and glucose. Insulin does not cross the placenta, but glucose does, causing fetal hyperglycemia and thereby fetal hyperinsulinemia (23, 24), which probably increases fetal growth in women with GDM. Also, women treated with insulin had higher fetal growth rates compared to both women with NGT and women with diet-treated GDM. We speculate that women with insulin-treated GDM have higher blood glucose, which might explain the resulting fetal overgrowth compared to the other groups. However, a distinct phenotype of for example higher BMI or genetic predisposition, could also account for these findings in women with insulin-treated GDM and insulin treatment can also be initiated because of excessive fetal growth. Other than glucose, increased supply of fatty acids and amino acids possibly also plays a role but perhaps owing more to higher BMI than the GDM itself (25).

We observed that the difference in fetal growth rate stagnated at approximately week 30 and declined faster in pregnancies affected by GDM after approximately week 33 (Fig. 2C), which could be due to the treatment following diagnosis in weeks 24 through 28.

In the developing fetus, insulin tends to stimulate abdominal growth (26), and abdominal circumference is a measure of fetal liver size and abdominal adiposity and has been found to be positively associated with weight-for-height *Z*-score in early childhood (27). We found fetal growth rate to be higher from late second trimester and abdominal circumference *Z*-scores to be increased already at weeks 19 through 24, similar to other studies suggesting that the increased fetal size in women with GDM is partly driven by an increased abdominal circumference (11, 13) and that especially abdominal circumference might be increased even before GDM diagnosis (28). This could be due to early fetal hyperinsulinemia before the diagnosis of GDM (29) because levels of fetal insulin in the amniotic fluid have been reported to be elevated already at week 16 in pregnancies in which women are later diagnosed with GDM (30). Also, a biphasic effect of diabetes on fetal growth has been shown, with decreased abdominal circumference in early second trimester followed by accelerated in utero growth in pregnancies affected by GDM (31), suggesting that the pattern of growth is of importance. Others have found increased EFW in women with GDM from week 28 (11), similar to our findings, but decreased EFW in week 24 has also been reported (32). Contrary to these findings, Zou et al found EFW to be higher in women with GDM only before week 24 but not in later pregnancy (12). This difference might be due to differences in diagnosis of GDM or in analysis method. We would expect the difference in EFW to be increased at the end of pregnancy because GDM is associated with large for gestational age birthweight and macrosomia (33, 34).

The effect of GDM on risk of overweight in the offspring attenuated when including fetal growth rate in the logistic regression, suggesting that fetal growth is a mediator of this association. In the Generation R study, EFW in second trimester, fetal weight gain from second to third trimester, third trimester to birth, and birthweight were associated with BMI in infancy (8). They found no association between the first trimester fetal size and postnatal growth measures, emphasizing that the critical time is probably in the second or third trimester. In the same cohort, there was no association with fetal growth and abdominal adiposity in the 2-year-old (35) or 6-year-old offspring, but fetal growth was associated with BMI (10). In the Project Viva Cohort, fetuses in the fourth quartile of EFW in second trimester had higher odds of becoming overweight compared to their counterparts in first quartile of EFW (7). Similarly, He et al found increased risk of overweight or obesity in 3-year-old children who had rapid growth in mid-pregnancy (6).

When adjusting for fetal growth rate in the association between maternal GDM and childhood overweight, the association attenuated by 15%, which can be interpreted as 15% of the effect of GDM on childhood overweight is mediated by fetal growth. We cannot, however, exclude that when adjusting for fetal growth, we might also unintentionally adjust for confounders that affect fetal growth (eg, placental function, genetics). If these factors also affect the risk of overweight, we might be overestimating the isolated effect of fetal growth.

When we adjusted for maternal pregestational BMI, the association between GDM and overweight in the offspring dissipated and we found only a small attenuation when adjusting for fetal growth rate. We believe that since pregestational BMI is highly associated with GDM, the interpretation of the models becomes difficult because of the risk of collinearity. On the other hand, Poprzeczny et al found increased fetal growth in women with overweight but no additional mediating effect by GDM, suggesting that the effect of high maternal BMI might saturate the effect on fetal growth without additional effect of GDM (36). The attenuation we observed in the association between fetal growth rate and overweight when adjusting for pregestational BMI could also suggest that other factors such as genetics or family lifestyle play a large role in the association. We believe that removing the effect of overweight is of less clinical importance since women with GDM are often overweight, making it difficult to separate the two.

A strength of this study is the large population and that there is a very high attendance to ultrasound screening from women from all parts of society and to the health nurse follow-up visits. Furthermore, by modelling fetal growth, we are able to incorporate both women with few and with multiple ultrasound measurements of fetal size in the study. Therefore, we believe we have obtained a broad representation of the population.

Our study is limited by the number of ultrasound scans performed for each individual. In Denmark, women are offered fetal ultrasound scans at weeks 12 and 20. However, the scan at week 12 is used to determine the gestational age, meaning that EFW is only obtained around week 20.

To create growth trajectories, we used additional scans performed on any indication. Women with GDM are routinely offered additional scans but bias might be introduced among the control group because the women with additional scans might have other conditions affecting fetal growth. When comparing women with additional scans performed, to women who received only standard care, birthweight *Z*-scores were very similar, but the rate of small-for-gestational-age was higher among women with additional scans, suggesting that fetal growth restriction could be the indication for some of the scans. However, because a large proportion of healthy women received additional scans and because they are similar to those receiving standard care on most parameters, we believe that the bias introduced, if present, is minimal and the results represent the background population (see Supplementary Table S4 (22)).

In our cohort, we experienced a large loss to follow-up after 5 years. This can partly be explained by the fact that to follow the children in the registries from fetal life to 5 to 9 years of age, the mothers had to live in Aarhus Municipality during pregnancy and stay afterwards for typically 6 years before starting school. Aarhus is a large university city in Denmark and living costs are generally high. Many families have children while living in Aarhus but later move to surrounding cites. This could potentially lead to selection bias of families of high socioeconomic status. Mobility probably does not completely explain the loss to follow-up but our supplementary analysis showed no major differences between those included in the cohort and those lost to follow-up; however, we lacked information on socioeconomic status.

In Denmark, risk-based screening rather than universal screening for GDM is used, which means some women with GDM might not be diagnosed and could therefore be in the control group. Because offspring of women with GDM had a higher risk of overweight, the bias introduced would probably be towards the null hypothesis. The GDM diagnosis was extracted from Astraia and therefore relies on registration of diagnosis by health care professionals. The care for pregnant women with GDM was managed by only a few medical doctors who have extensively registered diagnoses in Astraia during the study period. The advantage of this is that the risk of misclassification is very low.

Regarding GDM treatment, our data only comprised 37 women who received insulin treatment, which is reflected in the precision of the estimates comparing insulin- to diet treated-GDM.

In conclusion, we found that weekly fetal growth rate was higher in women with GDM compared to women with NGT. Children of mothers with GDM had a higher risk of being overweight. This risk attenuated when adjusting for fetal growth rate, meaning that part of the risk of overweight in offspring of mothers with GDM could potentially be explained by a mediating effect of higher fetal growth rate in the third trimester. However, prepregnancy BMI also had a considerable effect on the risk of overweight in the offspring, suggesting that other factors such as genetics and lifestyle play a large role in the risk of overweight in offspring affected by GDM.

The reason why some children develop overweight is still largely unknown, but our results suggest that fetal growth rate might be a contributor and potentially an early predictor in pregnancies affected by GDM.

## Data Availability

Restrictions apply to the availability of some or all data generated or analyzed during this study to preserve patient confidentiality or because they were used under license. The corresponding author will on request detail the restrictions and any conditions under which access to some data may be provided.

## Abbreviations

BMI: body mass index
EFW: estimated fetal weight
GDM: gestational diabetes
NGT: normal glucose tolerance
OGTT: oral glucose tolerance test
